# Huntington's Disease and Huntington's Disease‐like 2 (HDL2) in Martinique

**DOI:** 10.1002/mdc3.70379

**Published:** 2025-10-11

**Authors:** Ignacio Antolin‐Sanfeliz, Anna‐Gaelle Giguet‐Valard, Sophie Duclos, Cécile Cazeneuve, Chloé Angelini, Aïssatou Signaté, Russell L. Margolis, Cyril Goizet, Rémi Bellance

**Affiliations:** ^1^ Centre de Référence Caribéen des Maladies Neuromusculaires Rares, CHU de Martinique Fort‐de‐France France; ^2^ UF de Neurogénétique Moléculaire et Cellulaire, Département de Génétique, Hôpitaux Universitaires Pitié‐Salpêtrière, Assistance Publique—Hôpitaux de Paris Paris France; ^3^ Centre de Référence Maladies Rares “Maladie De Huntington Et Autres Chorées” and Centre de référence maladies rares “Neurogénétique”, Service de Génétique Médicale, Bordeaux University Hospital (CHU Bordeaux) and Univ. Bordeaux, CNRS, INCIA, UMR 5287, EPHE Bordeaux France; ^4^ Division of Neurobiology, Department of Psychiatry and Behavioral Sciences, and Department of Neurology John Hopkins University School of Medicine Baltimore Maryland USA

**Keywords:** Huntington's disease (HD), Huntington's disease type 2 (HDL2), neurodegenerative disorders, clinical characterization, epidemiology

## Abstract

**Background:**

Huntington's Disease‐like 2 (HDL2), caused by a CAG repeat expansion in *JPH3*, closely resembles HD. All reported HDL2 patients to date have some African ancestry. While both disorders exist in the Caribbean, their relative frequency and clinical characteristics remain largely unknown.

**Objectives:**

To characterize HD and HDL2 patients in Martinique.

**Methods:**

We retrospectively analyzed all HD and HDL2 patients evaluated over 20 years at a single neurology center in Martinique, collecting longitudinal clinical features, UHDRS scores, and repeat lengths.

**Results:**

In Martinique, combined HD and HDL2 minimum prevalence was 7.77/100,000. We ascertained 24 HD individuals, from 16 pedigrees, and 18 HDL2 individuals, from two pedigrees, one being the most extensive HDL2 pedigree yet reported. Because most HDL2 patients belong to a single large pedigree, the data must be interpreted with caution as familial clustering may introduce bias. HDL2 cases were predominantly male (83% vs. 45% in HD). Motor symptoms were the most frequent initial manifestation in both. Repeat length negatively correlated with estimated onset age in both diseases. Longitudinal motor (UHDRS‐TMS) and functional capacity (UHDRS‐TFC) scores in HDL2 revealed progressive worsening similar to HD. Inter‐ and intra‐familial clinical and genetic heterogeneity was obvious in both diseases. Anticipation was not exclusively reserved to paternal transmissions in HDL2.

**Conclusions:**

HDL2 is nearly as prevalent as HD in Martinique. The study reinforces the similarities between HD and HDL2 in genotype–phenotype correlation and disease course, while highlighting heterogeneity and germline instability in HDL2. Interpretation is limited by the small number of HDL2 families.

Huntington's disease (HD) is a neurodegenerative disorder clinically characterized by movement disorders, cognitive decline and psychiatric symptoms.^1^ HD is caused by a dominantly inherited CAG trinucleotide repeat expansion located, in the CAG orientation, in the first exon of the *HTT* gene encoding huntingtin. Penetrance is complete from 40 CAG triplets while there is a reduced penetrance between 36 and 39 CAG triplets. The intermediate range is considered between 27 and 35 and normal below 27.^2^ The repeat expansion causes progressive neuronal dysfunction and degeneration leading to progressive motor, psychiatric and cognitive signs, and ultimately to death within a mean of 20 years. The prevalence of HD highly depends on the population studied: from 1 to 7 individuals per million in Asian populations to 10.6–13.7 per 100,000 individuals in Western populations.^3^ In African populations, the prevalence remains unclear but seems considerably lower than in Caucasians.

Several other diseases may present with very similar features to HD although caused by different genetic factors.^4^ The disease most like HD, Huntington's Disease Like 2 (HDL2), was originally reported in 2001 by Margolis et al in an African‐American family.^5^ HDL2 is an autosomal dominant neurodegenerative disorder caused by a CAG trinucleotide repeat greater than 39 CAG at chromosome 16q24.3, falling with exon 2A of *JPH3* gene encoding junctophilin 3 (*JPH3*) on the sense strand, and within an open reading frame of *JPH3‐AS* on the antisense strand.^6^ Penetrance is complete above 40 repeats. Like HD, HDL2 is characterized by a triad of motor, cognitive and psychiatric signs and symptoms, with mid‐life onset and a progressive course over 10–20 years leading to death.^7^ Neuropsychological, imaging and neuropathological findings are also remarkably similar to HD; clinical and neuropathological experts cannot distinguish between the two diseases without a genetic diagnosis.^8^, ^9^, ^10^, ^11^, ^12^ HDL2 is extremely rare, with about 80 patients reported to date.

Martinique is an island in the French West Indies with a population of approximately 350,000. Originally inhabited by Carib Amerindians people, it was later populated by migrants from Europe, India, and predominantly Africa, through the transatlantic slave trade.

This African ancestry is especially relevant to HDL2, as the disease is found exclusively in individuals with a known or likely African ancestry and with a haplotype specific to individuals of African descent.^8^

While a few Caribbean cases of HDL2 have been reported, the clinical and epidemiological characteristics of HDL2 in the Caribbean have not been systematically examined. We therefore performed a systematic retrospective review of the medical records of HD and HDL2 patients diagnosed and followed between 2003 until 2023 in Martinique. We report the minimum prevalence of both diseases and describe their main clinical and genetic characteristics.

## Methods

### Subjects

All patients included in this study were seen at least once between 2003 and 2023 at the *Centre de Référence Caribéen des maladies neuromusculaires* (Caribbean Reference Center for Neuromuscular Diseases, CERCA), located on Martinique.^13^ HD and HDL2 were genetically confirmed in 42 individuals between 2003 and 2023: 24 individuals (57%) with HD from 16 unrelated pedigrees and 18 individuals (43%) from two pedigrees (one large and one small).

As CERCA is the only specialized medical facility on Martinique providing services to patients with rare neurological disorders, general practitioners are expected to systematically refer such patients to CERCA, resulting in a high level of ascertainment. When possible, genealogical trees were constructed for each patient. Clinical examinations were performed by experienced neurologists, with 38 patients receiving follow‐up visits every 6–12 months for a highly variable period of 1–20 years.

We performed a systematic retrospective review of the medical records of all CERCA HD and HDL2 patients; one HDL2 patient was previously described.^14^ Information collected from the records included gender; estimated age and signs at onset; age at genetic diagnosis; motor, cognitive and psychiatric features; trinucleotides repeat length; and, Unified Huntington's Disease Rating Scale (UHDRS)^15^ scores (Total Motor Score–TMS, and Total Functional Capacity–TFC) which were systematically collected on an annual basis after 2017. Motor, cognitive and psychiatric features were considered present if they were observed at any clinical assessment during follow‐up. Estimated age of onset was determined retrospectively based on clinical review of medical records and/or patient and family reports of first clear signs of disease.

This project was approved by the University Hospital of Martinique's IRB (2023/019).

### Genetic Analysis

Genetic analyses were performed at the French reference laboratory for neurogenetic disorders at University Hospital Pitié‐Salpêtrière, Paris, in all patients displaying clinical presentation suggestive of HD. Written consent was obtained from each patient prior to genetic testing in accordance with the French Bioethical law. Individuals with a known family history of HDL2 were first tested for the HDL2 mutation. Otherwise, individuals were first tested for the HD mutation, and if negative were then tested for the HDL2 repeat expansion. Repeat loci were amplified by PCR (polymerase chain reaction) and the number of repeats was determined by electrophoresis on an ABI‐3730 Genetic Analyzer (Applied Biosystems, Foster City, CA, USA) followed by analysis using Genemapper™ software (Applied Biosystems). For the most recent patients, the expanded repeats were sequenced using the Big‐Dye Terminator kit v2™ (Applied Biosystems) and analyzed with Sequencing Analysis software (Applied Biosystems), following conditions previously described.^14^

### Data Analysis

Statistical tests were performed in *SPSS Statistics* (version 25). When the data distribution was found to be normal, statistical values were reported as the mean and standard deviation. Non‐normal data were reported as the median and interquartile range. The association between estimated age at symptom onset and repeats length was performed by bivariate Pearson correlation. *R* ^2^ was used as the coefficient of determination for linear regression models. Prevalence estimation was performed based on the most recent available population census (2022).^16^ The incidence for each year was calculated using the population of each year from 2003 to 2022, and the resulting values were averaged to yield the average annual incidence.

## Results

### Trinucleotide Repeats Distribution

**Figure 1:**
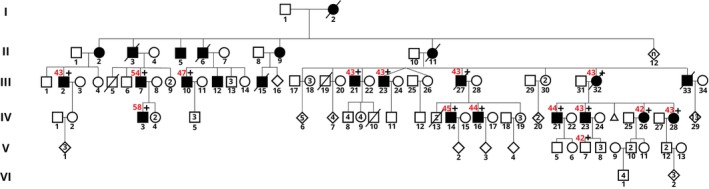
Pedigree 2 from the biggest HDL2 family in the cohort. In red: number of triplets. The identification of genetically diagnosed patient of the pedigree can be found in Table S1. Genetically diagnosed (+) are considered asymptomatic (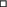) patients if they are clinically unaffected (no symptoms registered by the clinician).

Genetic confirmation was obtained for 24 HD patients (57%) and for 18 HDL2 patients (43%) from only two families, between 2003 and 2023. In HD, the repeats ranged from 40 to 61 CAG triplets (median: 43/IQR = 4), and in HDL2 from 42 to 58 CAG triplets (median: 43/IQR = 2) (see Table 1). All HDL2 patients were members of only two apparently unrelated pedigrees: a small pedigree including three genetically diagnosed patients and a large pedigree of more than 100 individuals including 15 genetically diagnosed patients (see Fig. 1). This constitutes strong limitations and potential bias of ascertainment in our studies.

**TABLE 1:** Symptoms manifestations, demographic and genetic characteristics by mutation group

		HD	HDL2*
n (%)		24 (57.1%)	18 (42.9%)
Sex; n (%)	Male	11 (45.8%)	15 (83%)
	Female	13 (54.2%)	3 (17%)
First symptom; n (%)	Motor	8/21 (38.2%)	9/15 (60%)
	Cognitive	2/21 (9.5%)	2/15 (13.3%)
	Psychiatric	4/21 (19%)	0/15 (0%)
	Motor + Cognitive	5/21 (23.8%)	3/15 (20%)
	Motor + Psychiatric	0/21 (0%)	0/15 (0%)
	Cognitive + Psychiatric	0/21 (0%)	0/15 (0%)
	Motor + Cognitive + Psychiatric	2/21 (9.5%)	1/21 (6.7%)
Motor involvement; n (%)	Chorea	22/23 (95.7%)	16/16 (100%)
	Dystonia	12/23 (52.2%)	5/16 (31.3%)
	Parkinsonism	7/23 (30.4%)	8/16 (50%)
Unrelated pedigrees; n		16	2 (15 individuals/3 individuals)

All patients	HD (n = 24)	HDL2* (n = 18)
Repeat length; Mdn (IQR)	43 (4)	43 (2)
Age onset (y) M; SD	49.6; 12.3	53.6; 13
Age genetic diagnosis (y) M; SD	54.7; 13.8	58.2; 13.3
Time onset–genetic diagnosis (y) M; SD	5.4; 3.7	7.3; 7.3

Patients with at least one UHDRS administration	HD (n = 16)	HDL2* (n = 12)
TMS scores; Mdn (IQR)	67 (27)	30 (33)
TFC scores; Mdn (IQR)	2 (3.5)	10 (8.75)
Repeat length; Mdn (IQR)	44 (5)	43 (2)
Age onset (y); Mdn (IQR)	47 (14)	54.5 (11)
Time onset to UHDRS testing (y) M; SD	11.2; 5.2	10.8; 5.6
Number of UHDRS testing (n); Mdn (IQR)	3 (2.75)	2 (1.75)
Age at UHDRS testing (y); Mdn (IQR)	62.2 (17.5)	64.8 (11.5)
Follow‐up time (y) M; SD	7.8; 4.5	6.1; 3.4
Number of visits; Mdn (IQR)	15.5 (20)	8 (5.75)
Average annual visits; Mdn (IQR)	2 (0.67)	1.44 (1.17)

*HDL2 data is collected from just two unrelated pedigrees.

Abbreviations: HD, Huntington's disease; HDL2, Huntington's disease like 2; IQR, Interquartile range; M, Mean; Mdn, Median; n, Number; SD, Standard deviation; TFC, Total functional score; TMS, Total motor score; UHDRS, Unified Huntington's disease rating scale; y, years.

### Clinical Features

Estimated age at onset, age at diagnosis and diagnosis delay are detailed in Table 1. Mean estimated age at onset was 49.6 years for HD and 53.6 years for HDL2. The HD and HDL2 patients showed a wide inter‐individual heterogeneity in UHDRS TMS and TFC scores. One HD patient and two HDL2 were still at a presymptomatic stage when evaluated. Detailed clinical and genetic data are depicted for all patients in Table S1.

The most common presenting features were motor abnormalities, observed in both HD (38.2%) and HDL2 (60%), followed by motor + cognitive (HD: 23.8%, HDL2: 20%). Psychiatric abnormalities were reported as presenting symptoms in HD (19%) but not in HDL2. Less frequently, cognitive impairment without motor or psychiatric abnormalities (HD: 9.5%, HDL2: 13.3%) or a combination of motor, cognitive, and psychiatric abnormalities (HD: 9.5%, HDL2: 6.7%) were the presenting features. No other combination of disease manifestations was reported at onset. Throughout the course of the disease, chorea was the most frequent motor feature (HD: 95%; HDL2: 100%). Parkinson's (30% HD; 50% HDL2) and dystonia (52% HD; 31% HDL2) were relatively common, though less so than chorea (Table 1). Five HD patients, but no HDL2 patients, reported suicide ideation (Table 2). When necessary, both HD and HDL2 patients were treated with antipsychotics (most commonly aripiprazole or pimozide) or a VMAT2 inhibitor (eg, tetrabenazine) with doses adjusted based on side effects and clinical benefit.

**TABLE 2:** Psycho‐behavioral manifestations in HD and HDL2

	HD	HDL2*	Total
Apathy	4/24 (16.7%)	2/18 (11.1%)	6/42 (14.3%)
Depression	8/24 (29.2%)	2/18 (11.1%)	10/42 (23.8%)
Anxiety	9/24 (37.5%)	2/18 (11.1%)	11/42 (26.2%)
Suicide Ideas	5/24 (20.8%)	0/18 (0%)	5/42 (11.9%)
Irritability	4/24 (16.7%)	6/18 (33.3%)	10/42 (23.8%)
Agitation	5/24 (20.8%)	4/18 (22.2%)	9/42 (21.4%)
Aggression	7/24 (29.2%)	4/18 (22.2%)	11/42 (26.2%)
Psychosis	6/24 (25%)	3/18 (16.7%)	9/42 (21.4%)
Disinhibition	8/24 (29.2%)	3/18 (16.7%)	11/42 (26.2%)
OCD symptoms	3/24 (12.5%)	2/18 (11.1%)	5/42 (11.9%)
Anosognosia	4/24 (16.7%)	1/18 (5.6%)	5/42 (11.9%)

*HDL2 data is collected from just two unrelated pedigrees.

Abbreviations: HD, Huntington's disease; HDL2, Huntington's disease like 2.

The use of UHDRS to quantify impairment of patients with HD or an HD‐like phenotype became part of standard clinical practice at CERCA for assessment of HD and HDL2 patients after 2016. UHDRS scores were collected at a median age of 62.2 (IQR: 17.5) in HD and 64.8 in HDL2 (IQR: 11.5). For HD patients, UHDRS were performed over a mean duration of 7.8 years (SD: 4.5), with a median of 3 (IQR: 2.75) assessments and a median of 2 visits/year (IQR: 0.67). For HDL2 patients, UHDRS were performed over a mean duration of 6.1 years (SD: 3.4), with a median of 2 (IQR: 1.75) assessments and a median of 1.44 visits/year (IQR: 1.17).

**Figure 2:**
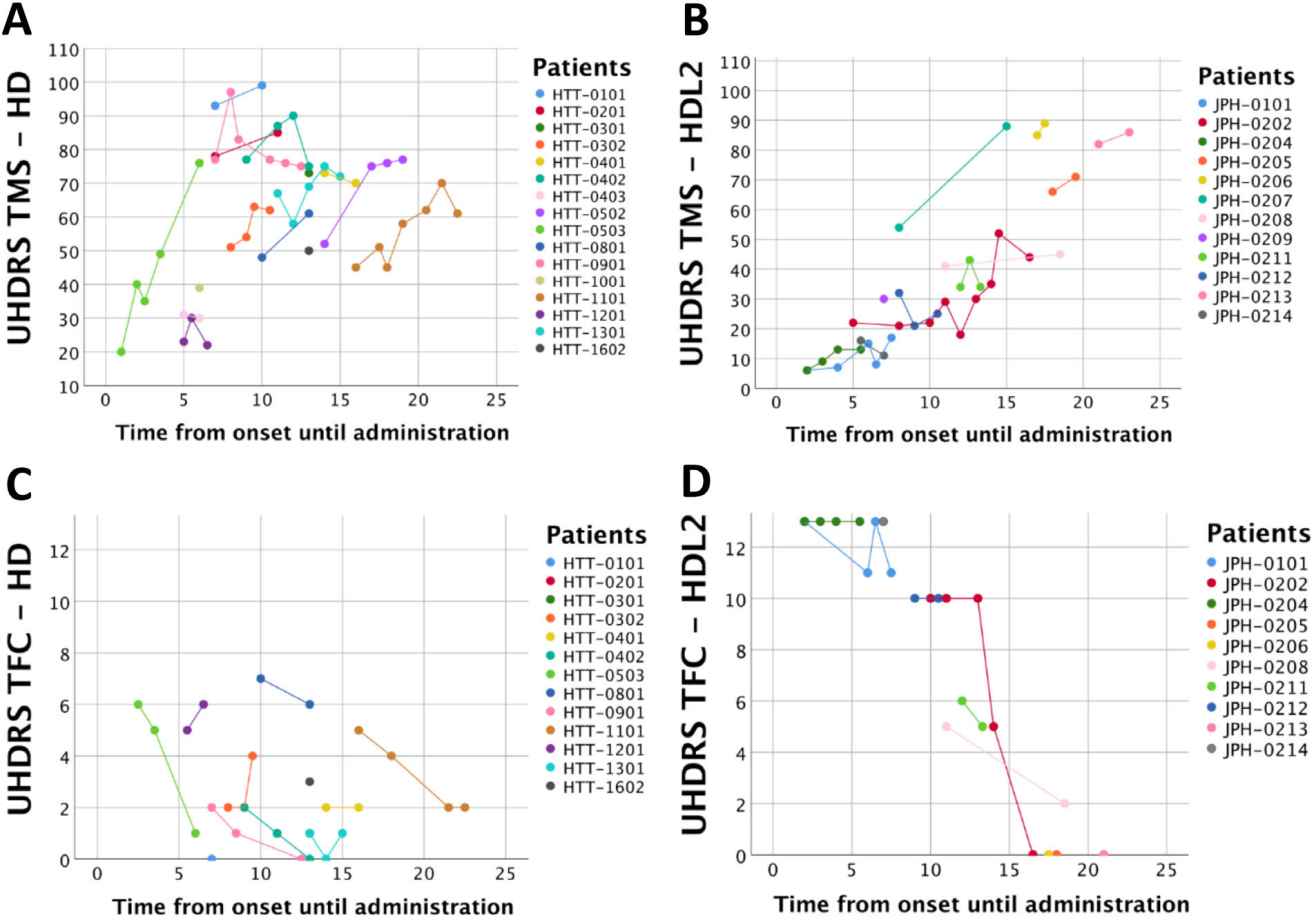
Scattered distribution of United Huntington's disease rating Scale—total motor scores and functional motor scores matched with the number of years from onset until the UHDRS was administered. Each dot represents a score administration and each color represents a patient. *Huntington's disease‐like 2 (HDL2) data is collected from just two unrelated pedigrees. (A) UHDRS‐TMS for HD group. (B) UHDRS‐TMS for HDL2 group. (C) UHDRS‐TFC for HD group. (D) UHDRS‐TFC for HDL2.

UHDRs motor scores progressively increased in both diseases, while TFC scores declined (Fig. 2). The highest TFC score recorded among HD patients was 7/13, indicating that all UHDRS scores were collected in patients past the early stage of disease. HDL2 patients maintained relatively stable TFC scores, except for one patient (JPH‐0202) who exhibited a rapid decline (Fig. 2). The HDL2 cohort showed a more diversified range of disease severity, ranging from early‐stages with normal TFC scores to advanced stages. At equivalent times from symptom onset, HD patients generally exhibited higher TMS scores than HDL2 patients, while TFC scores in HDL2 showed greater individual variability, with some cases of sharp decline.

### Phenotype–Genotype Correlations and Clinical Anticipation

**Figure 3:**
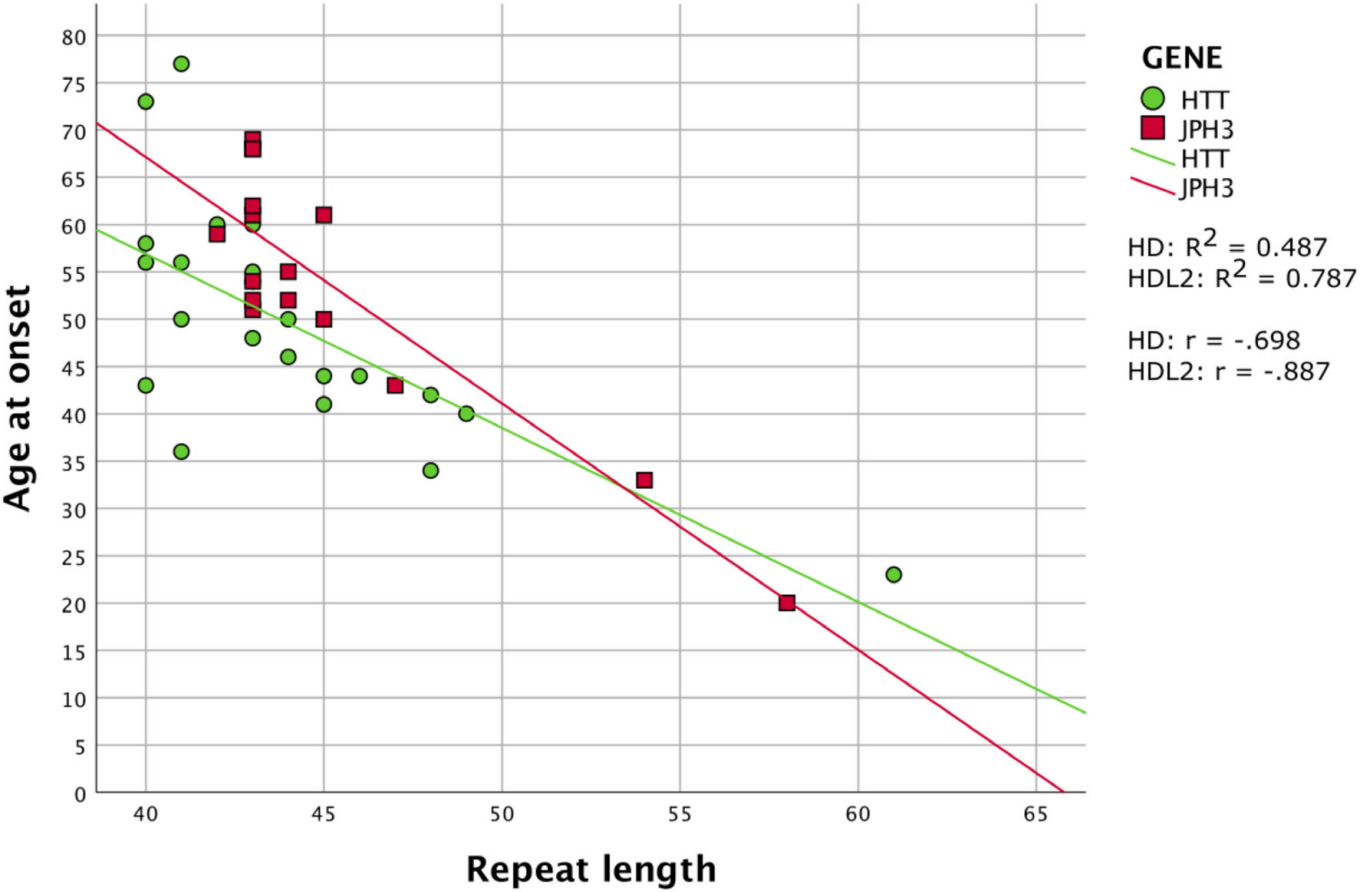
Scattered distribution by groups (HD and HDL2) of the repeat length and the age at onset. The graphic includes fit lines for each group, the Pearson correlation coefficient (r) and their respective coefficient of determination (*R* ^2^). *Huntington's disease‐like 2 (HDL2) data is collected from just two unrelated pedigrees. Linear regression equations: *y* = 1.32E2‐1.84*x for HD and *y* = 1.71E2‐2.6*x for HDL2.

The length of CAG repeats was negatively correlated with estimated age of onset in HD (*P* = 0.001/*r* = −0.698/*R* ^2^ = 0.487) and in HDL2 (*P* = 0.001/*r* = −0.887/*R* ^2^ = 0.787) (Fig. 3). HD individuals carrying the smallest expansion (40 CAG) developed symptoms at age 42, 58, and 73, respectively, showing large clinical heterogeneity, although the onset was much earlier (age 23) for the patient who carried the largest expansion (61 CAG). In HDL2, estimated onset was reported as early as 20 years in patient JPH‐0205 carrying the largest expansion (58 CAG) (Table S1).

Within the small HDL2 family, the repeat length expanded from 43 triplets in the father to 45 triplets in both sons, without clinical anticipation. In the large HDL2 family, repeat length ranges from 42 to 58 triplets, demonstrating clear intergenerational repeat length instability, evident in both generations III and IV. Eight parent‐offspring transmissions of repeat length were determined. The largest increase was from 54 triplets to 58 triplets (father III.7 [JPH‐0215] to son IV.3 [JPH‐0205]), associated with a 13‐year earlier age of onset. Three other expansions were of 1 or 2 triplets in length, of uncertain significance given the normal variation in PCR‐based repeat length determination: father III.27 (JPH‐0206) (43 triplets) transmitting to son IV.14 (JPH‐0210) (45 triplets) and son IV.16 (JPH‐0212) (44 triplets), and mother III.32 (JPH‐0201) (43 triplets) to son IV.21 (JPH‐0202) (44 triplets) (unexpectedly associated with a 17‐year earlier age of onset). Mother III.32 (JPH‐0201) also transmitted an unchanged allele to son IV.23 (JPH‐0204). Two contractions of one triplet were also noted: mother III.32 (JPH‐0201) (43 triplets) to daughter IV.26 (JPH‐0214) (42 triplets), and father IV.23 (JPH‐0204) (43 triplets) to son V.7 (JPH‐0203) (42 triplets).

### Epidemiologic Data

The gender distribution was balanced in HD with 11 males (45.8%) and 13 females: (54.2%). A strong male predominance was observed in HDL2 with 15 males (83%) and three females (17%) (see Table 1).

In 2023, 16 HD and 11 HDL2 patients live on Martinique, yielding a minimum prevalence of HDL2 of 3.16/100,000 people and a minimum prevalence of HD of 4.61/100,000, with a combined minimum prevalence of 7.77/100,000 people. These estimates are based on currently living, genetically confirmed individuals and do not account for undiagnosed cases. Given that all HDL2 are from two families (and the majority from one extended pedigree), these figures should be interpreted cautiously and do not necessarily reflect prevalence among unrelated individuals. The distribution of HD and HDL2 prevalence in 2023 are shown in Table 3.

**TABLE 3:** Distribution and prevalence of HD and HDL2 in Martinique from 2003 to 2023 and estimated minimum prevalence in 2023

HD and HDL2 distribution and prevalence								
	2003–2023			2023				
Group	HD, n (%)	HDL2*, n (%)	Total, n (%)	HD	HDL2*	Minimum Prevalence (per 100,000)		
						HD	HDL2*	Combined
Total	24 (57%)	18 (43%)	42 (100%)	16	11	4.6	3.16	7.77

Distribution of HD and HDL2 by population subgroups in Martinique from 2003 to 2023, and estimated minimum prevalence per 100,000 people in 2023.^16^ *HDL2 data is collected from just two unrelated pedigrees.

Abbreviations: HD, Huntington's Disease; HDL2, Huntington's disease like 2; n, number.

## Discussion

We describe for the first time the genetic, epidemiological and clinical characteristics of patients with HD (n = 24) and HDL2 (n = 18) in Martinique, collected retrospectively over a period of 20 years of assessment and follow‐up in a single specialty center. The unique circumstances of medical care in Martinique, where general practitioners and neurologists refer all patients with complex or suspected genetic neurological diseases to a single center, CERCA, suggests a high level of ascertainment. However, some affected individuals remain undiagnosed due to asymptomatic status or reluctance to seek clinical care. After South Africa, the HDL2 group in our study is the largest series of HDL2 patients ever recorded from a specific region,^17^ and the larger of the two HDL2 pedigrees described here is the largest HDL2 pedigree reported to date. The relatedness of most HDL2 patients represents a major potential source of bias, limiting the generalization of these findings.

### Epidemiology

In Martinique, both HD (4.6/100,000) and HDL2 (3.16/100,000) have high minimum prevalence rates, considering that populations of mixed or predominantly African ancestry typically show lower prevalence of these diseases.^18^ Additionally, because HDL2 has only been found in individuals with African ancestry, and our calculation is based on the total population of Martinique—including individuals of non‐African descent—the actual burden of HDL2 in the at‐risk population may be underestimated. However, since all HDL2 cases belong to just two families, the observed prevalence may also overestimate the rate of the disease among unrelated individuals. Together, these factors suggest the need for caution when interpreting prevalence figures.

The frequency of reported HDL2 cases in the island is high compared to neighboring regions Mexico,^19^ Brazil,^20^, ^21^ and Venezuela,^22^ where relatively few cases have been reported. The potential bias of our study based on a case series of HDL2 patients who are largely related to the same single family needs to be emphasized. The combined prevalence of HD and HDL2 in Martinique (7.77/100,000) appears high but this calculation includes multiple affected individuals from only two HDL2 families and should not be directly compared to population‐based studies in unrelated individuals such as those from South Africa (black [0.25/100,000], white [5.1/100,000], and mixed [2.10/100,000]).^17^

### Demographics Characteristics

The HD group had a similar male to female ratio. However, 83% of the HDL2 cohort were men. A systematic review of HDL2 previously found a sex ratio of 1.9:1 (M:F).^23^ We were unable to determine the cause of our cohort's sex disparity; it is possible that the mutation differentially affects conception, implantation, or early survival; that social dynamics on Martinique have led to differential ascertainment of men and women; or that the results reflect a chance finding that will not be confirmed in other populations.

### Clinical Characteristics

Motor symptoms alone were the most common signs of disease onset in both HD and HDL2, followed by a combination of motor and cognitive symptoms. However, since our clinic specializes in neurological and neuromuscular diseases, it is possible that motor features attracted more attention than psychiatric or cognitive signs and symptoms. Chorea was the most common symptom in both HD and HDL2, as observed in previous reports. Similarly, parkinsonism and dystonia were less prevalent.^24^, ^25^, ^26^ Parkinsonism may represent, at least partially, side effects of medication used to treat chorea or psychiatric symptoms. However, both HD and HDL2 can be characterized by prominent^27^, ^28^ or isolated parkinsonism.^29^

The UHDRS TMS and TFC scores demonstrated, as anticipated, progressive worsening of HD over time, with some fluctuation in motor scores from visit to visit.^30^ Importantly, our findings show, as other cross‐sectional studies, similar motor and functional deterioration over time in HDL2.^23^ Although the sample is small and mostly from one family, visual trends suggest that some HDL2 patients may experience steep functional deterioration compared to. These observations should be interpreted cautiously.

Large scale studies of HD, including ENROLL‐HD and REGISTRY, indicate that females with HD manifest a slightly more severe phenotype, and undergo a more rapid rate of progression in motor and functional scores than males.^31^, ^32^ If this sex‐based difference in severity and course is present in HDL2, it is possible that the limited representation of women in the HDL2 group might bias our results toward a modestly less severe phenotype.

Despite a study indicating that neuropsychiatric features in HD and HDL2 are generally similar, with early severity in HDL2 linked to motor decline,^10^ the present study identified more patients with neuropsychiatric features in HD than in HDL2. Moreover, the absence of such features in the HDL2 cohort prevented the evaluation of their early‐stage severity.

Our findings are consistent with previous findings that HDL2 is the condition that most closely resembles HD.^8^ It would be valuable to more precisely compare the course of HD and HDL2 with a prospective longitudinal study.

### Genetic Characteristics

Consistent with the previous literature, we found a strong negative correlation between age at onset and the length of trinucleotide repeats in HD^1^, ^33^, ^34^ and HDL2,^8^, ^23^, ^24^ and the relationship was similar in the two diseases (Fig. 3, Table S1). The larger of the two HDL2 pedigrees that we ascertained is the largest HDL2 pedigree yet reported in the literature, with 15 genetically diagnosed relatives over five generations. The high range in repeat lengths, from 42 to 58, provides clear evidence, not previously available, of repeat length variation in vertical transmission. Paternal inheritance has been tentatively associated with germline expansions and clinical anticipation in HDL2.^35^ In our study, decreases in age of onset from parent to child were reported both in a paternal expansion of four triplets and in a mother contraction of one triplet. Though this might suggest anticipation, such interpretations must be cautious. Increases of 1–4 repeats are relatively modest and are commonly observed in intergenerational transmissions in both HD and HDL2. Moreover, age of onset estimates is often imprecise and may be influenced by increased diseases awareness in subsequent generations.

In a study made in Venezuela, four families carried with the mutation the same haplotype typical of African populations.^22^ It would be interesting for the next study to compare if the two HDL2 pedigrees in Martinique share the same African haplotype. As the routes of population migrations due to transatlantic slave trade are similar between Martinique and Venezuela, it is possible that they also share a common ancestry origin of the mutation.

### Limitations

The main limitation of our study is the fact that most HDL2 patients belong to a single extended family, which limits the generalizability of epidemiological and clinical findings. Also, the limited sample size makes interpretation of the findings challenging; defining a clear clinical trajectory in HD has required analysis of hundreds to thousands of patients, a sample size not possible in HDL2, leading to some inconsistent findings across studies. Ascertainment of HD patients in the present study was likely limited, as suggested by the small size of the pedigrees that were examined. High UHDRS TMS and TFC scores were reported in individuals with HD who had a relatively recent onset. These scores, indicating advanced disease stages just a few years after onset, suggest that the reported onset may be delayed, distorting the findings, as the motor and functional scores don't align with expected diseases progression. Reported symptoms were restricted to those observed during follow‐up, and hence, additional symptoms may manifest in the future. As a retrospective study, there is considerable inter‐patient variability in the quality, quantity, and reliability of the different variables. Finally, the HDL2 group had an important female under‐representation which could bias the results.

## Conclusion

The study highlights clinical and genetic features of HD and HDL2 in Martinique, and confirms the similarity of HD and HDL2. HDL2 prevalence in Martinique is high although a very big pedigree bias these figures. Limitations in the results and their interpretation need to be considered as HDL2 data comes from only two families. HDL2 motor function and functional capacity deteriorated over time, following a similar trajectory as HD. HD and HDL2 clinical features are heterogeneous, even within the same family. Trinucleotide repeat length inversely correlated with disease onset in both HD and HDL2, with an unexplained excess of males with HD. Analysis of a large HDL2 pedigree revealed repeat length instability during vertical transmission, although changes were typically small, and the clinical implications remain uncertain.

## Author Roles

(1) Research Project: A. Conception, B. Organization, C. Execution; (2) Statistical Analysis: A. Design, B. Execution, C. Review and Critic; (3) Manuscript: A. Writing of the first draft, B. Review and Critique.

I.A.S.: 1A, 1B, 1C, 2A, 2B, 3A, 3B.

A.G.G.V.: 1A, 1B, 1C, 2A, 2C, 3A, 3B.

S.D.: 1A, 1C, 2C, 3B.

C.C.: 1C, 2C, 3B.

C.A.: 2C, 3B.

A.S.: 1C, 2C, 3B.

R.L.M.: 2A, 2C, 3B.

C.G.: 1A, 1B, 1C, 2C, 3B.

R.B.: 1A, 1B, 1C, 2C, 3B.

## Disclosures

**Ethical Compliance Statement:** This project was approved by the University Hospital of Martinique's IRB (2023/019). Patients were systematically informed during their consultations that their clinical data could be used for research purposes and written informed consent was systematically collected at the time of the sampling for the genetic diagnosis following French Bioethical Law. They were given the opportunity to opt out at any time if they did not wish for their data to be used in research. We confirm that we have read the Journal's position on issues involved in ethical publication and affirm that this work is consistent with those guidelines.

**Funding Sources and Conflicts of Interest:** AGGV Received financial support from Girci Soho for Huntington's related research; RLM received financial support for research activities from ABCD Charitable Fund and Abramson Fund; CG received financial support for clinical research activities from Registry of the European Huntington Disease Network (EHDN) and Enroll HD; the rest of the authors have no financial disclosure or conflict of interest to report related to the study.

**Financial Disclosures for the Previous 12 Months:** AGGV received grants from FILNEMUS and Girci Soho; SD received honoraria for lectures from Biogen and Alexion; AS received honoraria for lectures from Alnylam; RLM received funding from the National Institute of Mental Health (NIH MH120550, NIH MH128715, NIH MH129277, NIH MH015330) and the National Institute of Neurological Disorders and Stroke (NIH NS119671, NIH NS122756, NIH NS125350); CG received consulting fees from Sanofi, Amicus, and Biogen; honorarium from Chiesi for participation in an advisory board; honoraria for lectures from Sanofi, Biomarin, Biogen; financial support for research activities from Agence Nationale de la Recherche (ANR), Biomarin; inscriptions and travels for congresses were funded by Takeda and Chiesi; RB organizes a rare diseases congress sponsored by Alnylam, Biogen, Alexion, Argenx, Chiesi, Novartis, PTC therapeutics, Orphalan and Sanofi; the rest of the authors have no financial disclosure to report.

## Supporting information


**TABLE S1.** Descriptive data of patients with a positive HD and HDL2 diagnosis. The code of the patient is composed by four numbers, the first two refer to the family and the second to the individual. “Code Genealogy” refers to the identification of the patient in Figure 1. Cogn., cognitive; F, female; HTT, Huntingtin; JPH3, Junctophilin‐3; M, male; MRI, magnetic resonance imaging; N.A., not available; Psych., psychiatric; UHDRS, United Huntington's disease rating scale.

## Data Availability

The data that supports the findings of this study are available in the supplementary material of this article.
