# Supplementary material for: Huntington's Disease and Huntington's Disease‐like 2 (HDL2) in Martinique
Source: Mov Disord Clin Pract. 2025 Oct 11;13(3):673–82. doi: 10.1002/mdc3.70379 (PMC13042424; doi:10.1002/mdc3.70379)
Supplement: Supplementary file 1 — TABLE S1. Descriptive data of patients with a positive HD and HDL2 diagnosis. The code of the patient is composed by four numbers, the first two refer to the family and the second to the individual. “Code Genealogy” refers to the identification of the patient in Figure 1. Cogn., cognitive; F, female; HTT, Huntingtin; JPH3, Junctophilin‐3; M, male; MRI, magnetic resonance imaging; N.A., not available; Psych., psychiatric; UHDRS, United Huntington's disease rating scale. [file MDC3-13-673-s001.pdf]

SUPPLEMENTARY MATERIAL

- Supplementary Table 1. Descriptive data of patients with a positive HD and HDL2 diagnosis.

HTT and *JPH3* Gene Mutation

Code	Code Gene-alogy	Sex	Gene	Nº rep. mutation	Nº rep. normal	Age at genetic diagnosis	Estim-ated Age at onset	Onset to genetic diagnosis	Feature at onset	Motor troubles	Time onset to UHDRS (years)	UHDRS TMS	UHDRS TFC	Brain MRI (years after onset)
**HTT-0101**		F	**HTT**	43 ±1	15 ±1	55	54	1	Motor + Cogn.	Chorea + Dystonia	7	93	0	Diffuse atrophy and hyperintense punctiform images (1)
											10	99	-	
**HTT-0201**		F	**HTT**	49 ±1	17 ±1	41	40	1	Motor + Cogn.	Chorea	7	78	-	N.A.
											11	85	1	
**HTT-0301**		F	**HTT**	43 ±1	17 ±1	65	55	10	Motor + Cogn. + Psych.	Chorea + Parkinsonism + Dystonia	13	73	1	Cortical atrophy and small ventricular dilatation (3)
**HTT-0302**		F	**HTT**	43 ±1	17 ±1	66	60	6	Motor	Chorea + Dystonia	8	51	2	N.A.
											9	54	2	
											9.5	63	-	
											10.5	62	-	
**HTT-0401**		M	**HTT**	48 ±1	15 ±1	42	34	8	Motor	Chorea	14	73	2	Atrophy of the caudate nucleus (?)
											16	70	2	
**HTT-0402**		M	**HTT**	48 ±1	15 ±1	44	42	2	Motor + Cogn.	Chorea	9	77	2	Very marked cortical atrophy of the parietal lobes and atrophy of the caudate nucleus (?)
											11	87	1	
											12	90	-	
											13	75	0	
**HTT-0403**		M	**HTT**	46 ±1	15 ±1	50	44	6	Motor	Chorea + Dystonia	5	31	-	N.A.
											6	30	-	
**HTT-0501**		M	**HTT**	41 ±1	17 ±1	86	77	9	Cognitive	Chorea	-	-	-	N.A.
**HTT-0502**		M	**HTT**	45 ±1	16 ±1	50	44	6	Motor + Cogn. + Psych.	Chorea + Akinesia + Dystonia + Parkinsonism	14	52	-	Normal (2)
											17	75	-	
											18	76	-	
											19	77	-	
**HTT-0503**		F	**HTT**	61 ±1	22 ±1	24	23	1	Psychiatric	Chorea + Parkinsonism	1	20	-	N.A.
											2	40	-	
											2.5	35	6	
											3.5	49	5	
											6	76	1	
**HTT-0601**		F	**HTT**	41 ±1	15 ±1	44	36	8	Motor	Chorea	-	-	-	N.A.
**HTT-0701**		M	**HTT**	42 ±1	17 ±1	62	N.A.	N.A.	N.A.	Chorea	-	-	-	N.A.
**HTT-0702**		F	**HTT**	41 ±1	N.A.	66	56	10	Motor + Cogn.	Chorea	-	-	-	N.A.

**HTT-0801**		F	**HTT**	45 ±1	15 ±1	43	41	2	Motor + Cogn.	Chorea + Dystonia	10	48	7	N.A.
											13	61	6	
**HTT-0901**		F	**HTT**	42 ±1	18 ±1	64	60	4	Motor	Chorea + Dystonia	7	77	2	N.A.
											8	97	-	
											8.5	83	1	
											10.5	77	-	
											11.5	76	-	
											12.5	75	0	
**HTT-1001**		M	**HTT**	43 ±1	19 ±1	54	48	6	N.A.	Chorea + Dystonia	6	39	-	N.A.
**HTT-1101**		M	**HTT**	40 ±1	22 ±1	64	58	6	Motor	Chorea + Dystonia + Parkinsonism	16	45	5	T2 hyposignal of lenticular nuclei and caudate nucleus head (14)
											17.5	51	-	
											18	45	4	
											19	58	-	
											20.5	62	-	
											21.5	70	-	
											22.5	61	2	
**HTT-1102**		F	**HTT**	40 ±1	15 ±1	42	43	-1	Cognitive	ASYMPTOMATIC	-	-	-	Normal (0)
**HTT-1201**		M	**HTT**	44 ±1	23 ±1	49	46	3	Psychiatric	Chorea + Dystonia + Mild Parkinsonism	5	23	-	Cerebellar atrophy (2) / Diffuse atrophy (5)
											5.5	30	5	
											6.5	22	6	
**HTT-1301**		F	**HTT**	44 ±1	17 ±1	61	50	11	Psychiatric	Chorea + Dystonia + Parkinsonism	11	67	-	Diffuse atrophy with frontal predominance. Bilateral atrophy of the putamen and caudate nucleus (11)
											12	58	-	
											13	69	1	
											14	75	0	
											15	72	1	
**HTT-1401**		M	**HTT**	45 ±1	18 ±1	43	N.A.	N.A.	N.A.	ASYMPTOMATIC	-	-	-	N.A.
**HTT-1501**		F	**HTT**	40 ±1	19 ±1	78	73	5	Motor	Chorea + Dystonia + Parkinsonism	-	-	-	Ischemic area in left posterior junction suggesting vascular sequela (5)
**HTT-1601**		M	**HTT**	41 ±1	19 ±1	52	50	2	Motor	Chorea	-	-	-	N.A.
**HTT-1602**		F	**HTT**	40 ±1	15 ±1	68	56	12	Psychiatric	Chorea	13	50	3	N.A.
**JPH-0101**		M	**JPH3**	45 ±1	15 ±1	50	50	0	Cognitive	Chorea	2	6	13	N.A.
											4	7	-	
											6	15	11	
											6.5	8	13	
											7.5	17	11	
**JPH-0102**		M	**JPH3**	43 ±1	N.A.	59	51	8	Motor	Chorea	-	-	-	Subcortical atrophy (?)
**JPH-0103**		M	**JPH3**	45 ±1	16 ±1	41	N.A.	N.A.	N.A.	ASYMPTOMATIC	-	-	-	N.A.
**JPH-0201**	III-32	F	**JPH3**	43 ±1	16 ±1	74	69	5	Motor + Cogn. + Psych.	Chorea + Parkinsonism	-	-	-	N.A.

**JPH-0202**	IV-21	M	**JPH3**	44 ±1	15 ±1	57	52	5	Motor + Cogn.	Chorea + Mild Dystonia + Parkinsonism	5	22	-	N.A.
											8	21	-	
											10	22	10	
											11	29	10	
											12	18	-	
											13	30	10	
											14	35	5	
											14.5	52	-	
											16.5	44	0	
**JPH-0203**	V-7	M	**JPH3**	42 ±1	14 ±1	32	N.A.	N.A.	N.A.	ASYMPTOMATIC	-	-	-	N.A.
**JPH-0204**	IV-23	M	**JPH3**	43 ±1	15 ±1	63	61	2	Motor	Mild Chorea	2	6	13	Normal (0) / Diffuse atrophy (6)
											3	9	13	
											4	13	13	
											5.5	13	13	
**JPH-0205**	IV-3	M	**JPH3**	58 ±1	16 ±1	31	20	11	Motor + Cogn.	Chorea + Dystonia + Parkinsonism	18	66	0	Cerebellar atrophy associated with signs of ischemia (related with drug abuse) and anterior bilateral hyperintensity images through the white matter to the caudate nucleus (13)
											19.5	71	-	
**JPH-0206**	III-27	M	**JPH3**	43 ±1	13 ±1	78	68	10	N.A.	Chorea	17	85	-	N.A.
											17.5	89	0	
**JPH-0207**	III-21	M	**JPH3**	43 ±1	19 ±1	75	68	7	Motor	Chorea + Parkinsonism	8	54	-	N.A.
											15	88	-	
**JPH-0208**	III-2	M	**JPH3**	43 ±1	14 ±1	64	54	10	Motor	Chorea + Dystonia	11	41	5	N.A.
											18.5	45	2	
**JPH-0209**	IV-28	F	**JPH3**	43 ±1	15 ±1	57	62	-5	Motor	Chorea + Parkinsonism	7	30	-	Diffuse subcortical atrophy (6)
**JPH-0210**	IV-14	M	**JPH3**	45 ±1	15 ±1	61	61	0	Motor	Mild Chorea	-	-	-	N.A.
**JPH-0211**	III-10	M	**JPH3**	47 ±1	19 ±1	54	43	11	Motor + Cogn.	Chorea + Dystonia	12	34	6	Diffuse enlargement of subcortical spaces marked by age and reduced size of caudate nuclei (11)
											12.3	-	4	
											12.6	43	-	
											13.3	34	5	
**JPH-0212**	IV-16	M	**JPH3**	44 ±1	15 ±1	59	55	4	Motor	Chorea + Parkinsonism	8	32	-	Normal (4)
											9	21	10	
											10.5	25	10	

**JPH-0213**	III-23	M	**JPH3**	43 ±1	16 ±1	71	52	19	Motor	Chorea + Dystonia + Parkinsonism	21	82	0	N.A.
											23	86	-	
**JPH-0214**	IV-26	F	**JPH3**	42 ±1	15 ±1	64	59	5	Cognitive	Mild Chorea + Parkinsonism	5.5	16	-	N.A.
											7	11	13	
**JPH-0215**	III-7	M	**JPH3**	54 ±1	13 ±1	58	33	25	Motor	Chorea	-	-	-	N.A.
